# A randomized controlled trial to improve psychological detachment from work and well-being among employees: a study protocol comparing online CBT-based and mindfulness interventions

**DOI:** 10.1186/s12889-020-09691-5

**Published:** 2020-11-16

**Authors:** Sara Tement, Saša Zorjan, Meta Lavrič, Vita Poštuvan, Nejc Plohl

**Affiliations:** 1 Department of Psychology, University of Maribor, Faculty of Arts, Koroska 160, SI-2000 Maribor, Slovenia; 2 Slovene Centre for Suicide Research, University of Primorska, Andrej Marušič Institute, Koper, Slovenia; 3 Department of Psychology, University of Primorska, Faculty of Mathematics, Natural Sciences and Information Technologies, Koper, Slovenia; 4 Science Institute, University of Maribor, Faculty of Education, Maribor, Slovenia

**Keywords:** Psychological detachment from work, Well-being, Burnout, Cognitive-behavioral intervention, Mindfulness intervention, Randomized controlled trial

## Abstract

**Background:**

The changing landscape of the work environment, which often encompasses expectations of employees being continuously available, makes it difficult to disengage from work and recover. This can have a negative impact on employees’ well-being, resulting in burnout, depression and anxiety, among other difficulties. The current study will test the effectiveness of two different online interventions (i.e., cognitive behavioral therapy; CBT and mindfulness-based stress reduction; MBSR) on employees’ psychological detachment, burnout and other variables related to general (e.g., life satisfaction) and work-specific (e.g., work engagement) well-being.

**Methods/design:**

The study is designed as a randomized control trial with two intervention groups (i.e., CBT, MBSR) and a waitlist control group. Participants will be full-time employees from a wide range of organizations from Slovenia, who report moderate difficulties with psychological detachment from work and burnout and are not receiving any other form of treatment. The online interventions will encompass 12 sessions over 6 weeks (2 sessions per week); each session will include 1) an active audio-guided session and 2) home assignments, accompanied by handouts and worksheets. The study outcomes (i.e., psychological detachment, burnout, general and work-specific well-being), potential mechanisms (i.e., work-related maladaptive thinking patterns, mindfulness) and moderators (e.g., supervisor support for recovery) will be assessed immediately before and after the interventions (pre and post measurement) and 3 months after intervention completion (follow-up). Additionally, participants will fill out questionnaires for the assessment of the central mechanisms and study outcomes each week.

**Discussion:**

We expect that the CBT-based intervention will lead to greater improvements in psychological detachment from work and burnout compared to the MBSR and the waitlist control group. Additionally, we expect that the CBT-based intervention will also lead to greater enhancement of both general and work-related well-being.

**Trial registration:**

https://doi.org/10.1186/ISRCTN98347361 [May 19, 2020].

## Background

Today’s work realities are clearly different from those decades ago. With work carried out at any given time anywhere and managerial expectations to be available anytime, anywhere, employees frequently find it difficult to psychologically “switch-off” their work during non-work time. Therefore, recovery from work is increasingly difficult to achieve [1]. At the same time, employee reports of stress, work-family conflict, and burnout are a cause for concern. A recent representative study found, for instance, that around 25% of EU employees report work-related stress for all or most of their work time (e.g., [2]). One in ten employees reports that their work does not allow them to spend as much time with their families as they would like [3]. Additionally, data from the 6th European Working Conditions Survey show that in several European countries more than 10% of employees always feel exhausted at the end of the working day [4]. At the societal and organizational levels, these widespread concerns notably hamper productivity and are associated with major healthcare-related costs [5].

A lack of psychological detachment from work and employee well-being are clearly interconnected. According to the effort recovery model [6], a period of unwinding after exposure to job demands is necessary to replenish energy levels and start the next work day in an optimal psychophysiological state. If recovery after work is hampered over a longer period, employees will eventually face negative consequences in terms of well-being. A crucial condition for recovery is psychological detachment from work, which refers to “an individual’s sense of being away from the work situation” ([7], p. 579). Detachment encompasses mentally and emotionally disconnecting from work and refraining from work-related tasks such as checking e-mails, reading work materials, or completing unfinished tasks [8, 9]. In the present study, the focus is on the negative side of psychological detachment where one worries about upcoming work tasks, ruminates about past work experiences, or feels pressured (either internally or externally) into work-related tasks during non-work time [9, 10]. Previous studies have found that poor detachment from work is positively associated with exhaustion, burnout, poor sleep, and work-family conflict [9, 11, 12]. At the same time, a lack of detachment is negatively linked to work engagement, life satisfaction, and other general well-being indicators.

Several individual-oriented intervention studies have shown that the ability to detach from work and unwind at home can be taught (e.g., [13]). In particular, interventions that include elements of cognitive-behavioral therapy (CBT) and mindfulness were found to help employees to psychologically detach from work during non-work time [13–18]. In addition, such interventions contribute to the prevention of stress, negative affect [13], anxiety, and depression [17, 19] as well as sleep disturbances [16, 17]. Beneficial effects of CBT and mindfulness interventions for employees are evident not only with respect to general well-being, but also for work-related outcomes such as exhaustion and burnout [17, 20], work engagement [21], job satisfaction [20], work-family conflict, and work-family balance [22, 23]. While previous studies suggest that both types of interventions can be effective, interventions with CBT elements show more pronounced positive effects in terms of employee well-being [24].

Nevertheless, the development and implementation of effective interventions for employees still raise many different questions. First, intervention research needs to expand the understanding of why CBT-based and mindfulness interventions are effective and which mechanisms are responsible for the beneficial effects [14, 25, 26]. Especially in organizational settings where time constraints, logistical hurdles, and managerial attitudes can limit the widespread use of such interventions, additional knowledge about mechanisms of change is crucial [25, 26]. In fact, it has previously been argued that psychological intervention studies should shift their focus from the effectiveness of the treatment to understanding the underlying mechanisms and, consequently, informing theory [27]. In order to provide cost- and time-effective evidence-based workplace interventions, a more nuanced understanding of what elements can be dropped from interventions, and what elements are decisive ingredients is needed [28]. Improved mindfulness skills, such as paying closer attention to one’s momentary activities instead of ruminating about the past or worrying about the future, were found to act as mechanisms of change (or mediators) of the intervention effects among employees [14, 17]. Other potential mechanisms which help to increase psychological detachment and employee well-being have yet to be considered.

Second, intervention research needs to determine what type of intervention or protocol is superior and whether the proposed mechanisms of change are general (i.e., operating across interventions), intervention-specific, or non-existent. As CBT-based and mindfulness intervention studies focus on one or the other type of intervention, direct comparisons of effects in a workplace context are not yet possible [26]. The majority of studies follow either the Mindfulness-Based Stress Reduction (MBSR [29]) or the Mindfulness-Based Cognitive Therapy (MBCT [30]) protocol [25]. Mixed protocols such as MBCT, which combines CBT methods and meditative mindfulness practices, make it additionally difficult to draw conclusions about which mechanisms are responsible for intervention effects. While the MBSR protocol especially focuses on achieving a purposeful awareness of the present moment through different mediations exercises, the MBCT protocol additionally includes exercises that help to identify maladaptive thinking patterns, reframe, and replace them. Adding another layer of complexity, MBSR and CBT may be effective because of similar underlying mechanisms. Similar to CBT, MBSR is also proposed to disconnect the maladaptive links between the way people think, feel, and behave (i.e., a desynchrony effect [31]). MBSR and CBT thus, both enable disengaging from maladaptive thinking patterns and, in turn, achieving cognitive change [32, 33]. On the other hand, CBT may also increase mindful awareness and thus address a similar change mechanism as MBSR [34].

Finally, a detailed understanding of different organizational, individual, and intervention design factors that have the potential to alter intervention effects is needed [25, 26]. Tentative evidence suggests that CBT- and mindfulness-based interventions may be more effective for white-collar employees [14]. Organizational variables such as managerial support for recovery [35], a climate that supports segmentation between work and home [36] as well as the level of unfinished tasks [37] may also represent essential boundary conditions. In cases where the workplace does not favor psychological detachment from work, and the amount of work tasks does not permit it, intervention effects on detachment and well-being outcomes are likely to be attenuated. Considering individual factors, studies have discussed the role of treatment-by-baseline interactions in which individuals with different baseline levels of the variables of interest show different reactions to interventions [16]. Particularly those individuals who are more at risk with respect to well-being indicators have the potential to gain more from the intervention. Lastly, intervention design factors such as the length of the intervention, commitment to homework assignments, and training demands (i.e., the complexity of homework assignments) are likely to play a role in attenuating and accentuating CBT- and mindfulness-based intervention effects [14, 15].

### Objectives and hypotheses

In order to advance CBT- and mindfulness-based intervention research for employees, the objectives of the present randomized control trial are: a) to evaluate the effectiveness of a MBSR intervention on psychological detachment and well-being indicators; b) to evaluate the effectiveness of a CBT-based intervention on psychological detachment and well-being indicators; c) to compare the strength of the effects of both protocols; d) to determine which mechanisms are responsible for the intervention effects (i.e., mediators) and test whether these mechanisms are general or intervention-specific; e) to explore boundary conditions (i.e., moderators) which attenuate or accentuate intervention effects. To determine the decisive ingredient in CBT-based and mindfulness interventions, the CBT protocol will predominantly be aimed at identifying and changing maladaptive thinking patterns and associated behaviors. The MBSR protocol, on the other hand, will predominantly include exercises that focus explicitly on increasing present moment awareness by using established exercises that aim to improve mindfulness practices [14, 38]. Although research has found that both CBT and MBSR protocols are effective with respect to employee well-being [14, 17, 18, 24], we tentatively assume that the CBT protocol will be superior for most of the studied outcomes as it is theory-driven, well-researched, and empirically supported for a wide range of conditions including moderate to severe symptoms of several psychological disorders [39]. It may also be more appropriate for targeting an important underlying factor contributing to poor detachment and burnout (i.e., maladaptive thinking patterns) [40]. Moreover, interventions that include some form of CBT exercises along with other exercises are found to be most effective [17, 18, 22, 41]. More precisely, we hypothesize that the CBT-based intervention will lead to higher levels of psychological detachment from work directly after the intervention (T2) and at follow-up (T3) compared to levels prior to the intervention (T1) and to MBSR and control groups. Additionally, we assume that the CBT-based intervention will lead to enhanced (general and work-specific) well-being directly after the intervention (T2) and at follow-up (T3) compared to levels prior to the intervention (T1) and to MBSR and control groups. In line with previous studies (e.g., [41]), a wider range of general (life satisfaction, depression, anxiety, stress, positive and negative affect, sleep quality) and work-related well-being (work engagement, workaholism, work-family conflict) outcomes will be assessed. Along with psychological detachment from work, burnout also represents a primary outcome as both interventions will be adapted to the work context and conditions of high job demands.

In line with one of the main objectives, we will focus on (work-related) maladaptive thinking patterns and mindfulness as mechanisms of change behind both protocols. Maladaptive thinking patterns are inflexible, unreasonable, and negatively-valenced cognitions that may guide one’s attention toward work and may lead to excessive preoccupation with work-related tasks [40]. Within CBT, these maladaptive thinking patterns are labeled as negative automatic thoughts or cognitive distortions. Mindfulness, on the other hand, is defined as an aware and non-judgmental state of mind in which one’s attention is directed toward the present moment [14, 38, 42]. Given that CBT is primarily aimed at altering maladaptive thinking patterns and MBSR at increasing trait mindfulness, we assume mechanisms of change during our intervention will correspond to the main goal of each protocol. In other terms, we hypothesize that work-related maladaptive thinking patterns will act as a mechanism through which the CBT-based intervention will contribute to higher psychological detachment from work and improved well-being, and mindfulness will act as a mechanism of change for the MBSR intervention. Since the proposed mechanisms may not be intervention-specific [33, 34], we will also test whether maladaptive thinking patterns and mindfulness act as mechanisms of change in MBSR and CBT-based intervention, respectively. As authors have argued that the proposed mediators and outcomes should be monitored not only at the end but also during the intervention [27], we will measure work-related maladaptive thinking patterns, mindfulness, psychological detachment, and burnout levels also on multiple occasions during the intervention. We assume that we will be able to establish a timeline between the proposed mediators and outcomes with a decrease of maladaptive thinking patterns and an increase of mindfulness across the intervention weeks associated with the primary outcomes of interest.

As CBT-based and MBSR interventions will most likely not be equally beneficial for all participants, we will further explore the moderating role of several organizational, individual, and intervention design factors. More precisely, we will focus on variables that have the potential to attenuate the effects of the intervention, primarily on psychological detachment and burnout (supervisor support for recovery, segmentation supplies, unfinished tasks, and time pressure). In line with previous studies, we will also consider compliance with intervention exercises, completion of homework assignments, and satisfaction with the interventions as potential moderating variables.

## Methods/design

### Study design

**Fig. 1:**
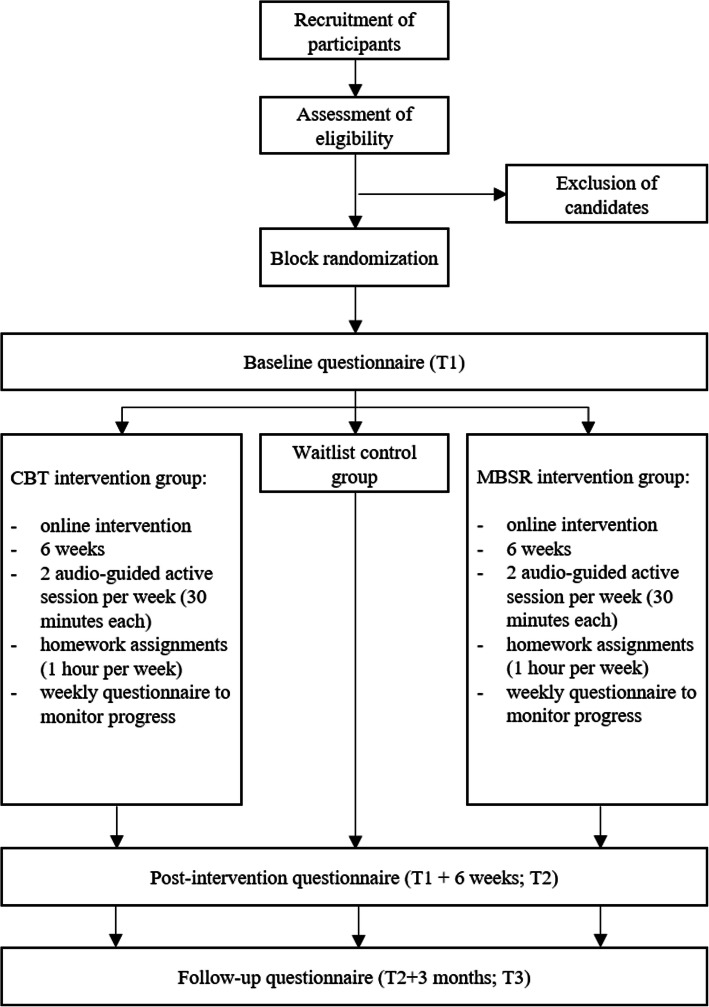
RCT intervention designs

The randomized controlled trial will follow the recommendations of the CONSORT (Consolidated Standards of Reporting Trials) group, which is considered a gold standard in clinical practice regarding devising interventions as well as their reporting [43]. The study design is depicted in Fig. 1. The study will take place between October 2020 and February 2021. In order to reach the target sample size, the second round of data collection will take place between January 2021 and May 2021, and additional rounds of data collection will be carried out if needed. Data collection in both intervention groups (i.e., CBT- and MBSR protocol) and the control group will be carried out immediately before (T1) and after the intervention (T2). During the 6-week intervention period, weekly questionnaires will be sent out to participants. The follow-up data collection will take place 3 months after the end of the intervention (T3). During T1, T2 and T3, participants will be asked to fill out questionnaires addressing all studied constructs, whereas the weekly questionnaire will only encompass the central mechanisms and primary study outcomes (i.e., work-related maladaptive thinking patterns, mindfulness, psychological detachment, and burnout). During the intervention period, participants from both intervention groups will receive e-mail notifications every Monday and Thursday with information about the assignments and exercises. The initial e-mail will include instructions on how to access the online platform with CBT and MBSR exercises. The notification every Monday will remind participants to fill out the weekly questionnaire before they start with the new exercise. All exercises from previous weeks will be visible on the online platform. Upcoming exercises will not yet be available to ensure that participants will not skip the activities or perform them in random order. The online platform will automatically monitor whether the participants followed the instructions and performed the respective exercises. In case of non-compliance, reminders will be sent out the following day. After the intervention, participants from the waitlist control groups will receive access to the online platform and all intervention materials, which they can perform in any order. They will receive weekly e-mails reminding them to perform the exercises and encouraging them to practice them. After the intervention, each participant will have the opportunity to request individualized feedback on his or her results.

### Participants and recruitment

The participants will be recruited from various organizations in Slovenia partnered with the leading institution and through different media channels. The intervention will also be advertised through university newsletters and webpages as well as a well-known Slovenian mental health platform. Interested participants will be able to apply for the study via an online form. Although we will not specifically exclude any professions, employees with knowledge-intensive jobs and high-stress occupations will be primarily targeted [44]. The recruitment stage will begin in August 2020. Following previous intervention studies in the workplace context (e.g., [17, 18]), participants will be pre-screened for eligibility. They will have to meet the following inclusion criteria: 1) being aged 18 or above, 2) working a minimum of 40 h per week and currently not on sick leave, 3) ability to commit (at least) 2 h per week to the online training, 4) ongoing internet connection and computer accessibility, 5) reporting difficulties with psychological detachment from work and burnout at least to a moderate extent. Participants undergoing other forms of treatment (psychotherapy, pharmacotherapy) or who have a clinical diagnosis of mental illness will not be eligible to participate. They will, however, receive additional information on relevant health care services and a link to mindfulness and CBT exercises they can practice on their own. Those eligible to participate will receive detailed information about the intervention and an online informed consent form. Participants who will commit to the study will be randomly assigned to one of the three groups. Following the approach by Querstret and colleagues [17], block randomization based on gender will be used. Participants will be randomized within blocks in such a way that an equal number of females and males are assigned to each of the groups because women exhibit more rumination and difficulties detaching from work at home (e.g., [17]). In order to control for expectations about potential benefits of the interventions, participants will be blinded to the intervention. Both the CBT-based and the MBSR intervention will be advertised as occupational stress-reduction interventions. The participants assigned to the waitlist control group will only be asked to participate in the study and fill out the questionnaires. They will also receive information regarding the delayed starting date of the training.

### Training sessions

Both interventions in the present study will take place online. Such a delivery has several advantages over more traditional on-site interventions, including the increased potential to attract people who might otherwise not decide to enroll in mental health interventions because of the stigma associated with them [45], people with busy schedules, and resulting time constraints [46] and people who live in rural and remote areas [47]. Perhaps even more importantly, an increasing body of literature suggests that online CBT-based and mindfulness interventions can improve outcomes related to work stress and well-being and may even be as effective as conventional face-to-face training (e.g., [15, 48–53]).

#### Preliminary study: selection of exercises

To address the issue of mixed protocols, which make it difficult to draw conclusions about the key mechanisms behind successful interventions, we conducted a preliminary study. The main objective of the preliminary study was to identify ten CBT and ten mindfulness exercises, which will represent the core elements of the intervention. In particular, we were looking for exercises that: 1) can be classified as (almost) purely CBT or mindfulness, 2) are suitable for the online environment, and 3) could be useful in terms of increasing psychological detachment and decreasing burnout (as these are our primary outcomes).

A total of 6 experienced psychologists (66.7% female, age: *M* = 34.00 years, *SD* = 7.85) participated in the preliminary study. Half of the participants (50.0%) had a doctoral degree. Participants’ knowledge of CBT (*M* = 4.00, *SD* = 0.63) and mindfulness (*M* = 3.83, *SD* = 0.75) - both self-reported on a scale from 1 (“*not knowledgeable at all*”) to 5 (“*very knowledgeable*”) - was high and relatively similar for both theoretical frameworks (the effect size, pertaining to the difference, was small; *d* = − 0.43). A third of the sample (33.3%) had previously designed and/or performed interventions that included CBT elements. The same percentage had previously designed and/or performed interventions that included mindfulness elements.

The participants were first given a short introductory instruction, which included basic information regarding both approaches (CBT and mindfulness). After answering demographic questions, participants were asked to read names and descriptions of 43 exercises (53.5% CBT, 46.5% mindfulness), with each exercise being accompanied by three questions. More specifically, participants were asked to rate whether the exercise was suitable solely for the CBT intervention, mindfulness intervention or was somewhere in between (a 5-point scale was used, with 1 meaning “*appropriate only for CBT*”, 5 meaning “*appropriate only for mindfulness*” and 3 meaning “*equally appropriate for both interventions*”). A similar question with a similar response format was asked regarding the intervention being suitable solely for an on-site/remote setting (a 5-point scale was used, with 1 meaning “*appropriate only for an on-site setting*”, 5 meaning “*appropriate only for a remote setting*” and 3 meaning “*equally appropriate for both settings*”). Lastly, participants were asked whether the exercise should be included in the intervention (1), could potentially be included in the intervention (2), or should not be included in the intervention (3). They were also given the opportunity to write down their comments for each exercise. It should be noted that the participants did not see the names of the exercises, which could completely reveal whether the exercises were mindfulness-oriented or CBT-oriented (e.g., “Mindful eating” was replaced by “Eating with awareness”).

Based on the results of the preliminary study, 20 exercises that met all three criteria were selected for our intervention. These exercises are listed and briefly described in Tables 1 and 2 below. The selected mindfulness exercises (Table 1) encompass all three main meditation practices (i.e., body scan, sitting meditation, mindful yoga [32, 69];) as well as some additional exercises, most of which stem from the work of Segal and colleagues [30]. On the other hand, the selected CBT exercises (Table 2), for example, introduce and address the three levels of cognition (i.e., core beliefs, dysfunctional assumptions, negative automatic thoughts) developed by Beck [70] as well as the ABC (activating event, belief, consequences) model developed by Ellis [58]. The selected activities could help participants understand their current ways of thinking and behaving and equip them with the tools that could change their maladaptive cognitive and behavioral patterns [69]. Most of the CBT exercises are adapted versions of worksheets published on the Psychology Tools website (https://www.psychologytools.com).

**Table 1:** Mindfulness exercises

Name of the exercise	Brief description of the exercise	CBT/Mindfulness*Me (IQR)*	On-site/Remote*Me (IQR)*	*Suitable/Not suitableMe (IQR)*
1. Body scan	The main idea of the exercise is (sequentially and non-judgmentally) bringing detailed awareness to each part of the body. Participants learn to keep their attention focused over a sustained period of time, which helps them develop concentration, calmness and flexibility of attention [30].	5.00 (0.00)	3.00 (0.75)	1.50 (1.00)
2. Mindful eating	The exercise involves a non-judgmental awareness of physical and emotional sensations while eating [54] or, in other words, a mindful (i.e., fully aware) approach to eating [55]. As eating is normally an “automatic act”, this exercise is a good illustration of the level to which we are often unaware of what is going on and an example of the changes that can occur if we slowdown and focus on simple acts [30].	5.00 (0.00)	3.00 (1.50)	1.00 (0.75)
3. Sitting meditation with focus on breathing	During the exercise, participants focus their attention(primarily) on their breathing, but also sounds in the environment, body sensations, and their stream of thoughts and emotions [30, 56]. The exercise helps participants to let go of the past and the future and to pay attention to the simple as opposed to analyzing the complex [30].	5.00 (0.00)	3.00 (0.75)	1.00 (0.00)
4. Paying attention during routine activities	The exercise encourages participants to choose one routine activity and make a deliberate effort to bring moment-to-moment awareness to the activity (e.g., brushing one’s teeth, taking a shower, taking out the garbage). By performing the exercise, participants start to realize that they can practice mindfulness by being present in all waking moments, no matter how ordinary and thus bring themselves back into the moment at any time [30].	5.00 (0.00)	3.00 (1.50)	1.00 (0.00)
5. Three-step breathing space	Participants learn how to become fully aware of their thoughts or feelings (Step 1: acknowledging what is going on), then, having acknowledged them, to move their attention to their breathing (Step 2: bringing attention to the breath), before expanding attention to the body (Step 3: expanding attention [30]). This form of mini-meditation helps participants gather a scattered mind and relate more skillfully to difficult emotions as they arise [55].	5.00 (0.75)	2.50 (1.00)	1.00 (0.00)
6. Mindfulness in everyday life	This is actually a compilation of a wide array of everyday informal mindfulness exercises, such as 1)focusing attention on breathing right after waking up, before leaving the bed, 2) using everyday sounds(e.g., birds singing) as a reminder of mindfulness, 3)being aware of bodily sensations when waiting in the line, … Such activities help an individual remember to be mindful in everyday life [55].	5.00 (0.00)	3.00 (1.50)	1.00 (0.00)
7. Mindful walking	The exercise takes the everyday activity of walking and uses it as a mindfulness practice. During the exercise, participants walk, knowing that they are walking and feeling the walking. It is a form of a meditation in motion and the focus is on maintaining moment-to-moment awareness of the sensations accompanying our movements, letting go of any thoughts or feeling about the sensations themselves. The exercise can be useful, because it enables people to feel more grounded, using the body as an anchor to the present moment [30].	5.00 (0.00)	2.50 (1.00)	1.00 (0.75)
8. Mindful movement	Mindful movement is based on yoga and falls under the category of body-based mindfulness exercises. During this exercise, participants are asked to perform movements such as stretching, raising up the arms, … The task is to pay attention to bodily sensations and notice which sensations are associated with each phase of the prescribed movements. This often enables participants to learn more about their bodies and to distinguish sensations in different parts of the body [30].	5.00 (0.00)	2.50 (1.00)	1.00 (0.75)
9. Breathing space: Adding the action step	This exercise is an upgraded version of the three-step breathing space exercise described above. It involves a fourth step –choosing what to do next in terms of activity. Activities that are pleasurable or give a sense of mastery may be particularly helpful. Whatever action is taken, the idea is to act mindfully. Deciding to act can help participants to regulate their mood and can be an important step toward improving health and well-being [30].	5.00 (0.75)	3.00 (1.50)	1.00 (0.75)
10. Staying present	This is a more general exercise that encourages participants to use their body as a way to awareness by, for example, staying mindful of their posture, paying attention to the sensations in their body at the moment, and being in their body as they move when they reach for something. The main idea is that patiently practicing feeling of what is there (and the body is always there) will help participants expand their awareness from times of formal meditation to living mindfully in the world [30].	5.00 (0.00)	3.00 (1.50)	1.50 (1.00)

**Table 2:** CBT exercises

Name of the exercise	Brief description of the exercise	CBT/Mindfulness*Me (IQR)*	On-site/Remote*Me (IQR)*	Suitable/Notsuitable*Me (IQR)*
1. Fact or opinion	The activity involves defining facts and opinions as well facilitating the differentiation between them. This is a critical skill in CBT, as it helps participants understand that their thought processes are not facts about the world, but their opinions or assumptions. The activity is a useful starting point when one wants to challenge the validity of negative thoughts [57].	1.00 (0.75)	3.00 (0.75)	*Me (IQR)* 1.00 (0.75)
2. ABC belief monitoring	ABC belief monitoring, originally developed by Ellis [58], is a type of functional analysis and thus explores the links between stimuli and responses. During the exercise, participants explore the antecedents or activating events (A), beliefs (B) and consequences (C). The exercise helps participants identify thoughts or beliefs which occur in a particular situation and identify the consequences of holding those beliefs. It is one the key exercises as it introduces the cognitive model.	1.00 (0.00)	3.00 (0.75)	1.00 (0.00)
3. Rumination diary	The rumination diary, similarly to other kinds of diaries used in CBT (e.g., worry diary), encourages participants to record (repetitive) thoughts and images. Participants are asked to record the triggers for the rumination as well as accompanying emotions, their ruminative content and the consequences of ruminations. They are also encouraged to think about what stopped the rumination [59].	2.00 (0.00)	3.00 (0.00)	1.00 (0.75)
4. Thought record	This exercise is one of the essential exercises in CBT. During the exercise, participants are encouraged to identify negative automatic thoughts, deepen their understanding of the relationship between thoughts and emotions, examine the evidence for and against a selected negative automatic thought, challenge a negative automatic thought and generate more realistic alternatives to a negative automatic thought. As such, the exercise helps participants evaluate their negative automatic thoughts for accuracy and bias [57, 60].	1.00 (0.00)	3.00 (0.75)	1.00 (0.00)
5. Thought distortion monitoring record	This activity is an expanded version of the thought record exercise described above. It is administered to help participants identify negative automatic thoughts, notice associations between events and cognitions, help clients understand the links between thoughts, emotions and body sensations, and, most importantly, begin to identify cognitive distortions in their thinking (e.g., jumping to conclusions, “should” statements, …). It is designed to increase participants’ awareness of biases or distortions in their thinking [57, 61].	1.00 (0.75)	3.50 (1.00)	1.00 (0.00)
6. Decatastrophizing	The exercise is a cognitive restructuring technique, normally used to reduce or challenge catastrophic thinking (also known as magnification). Participants are first asked to identify the catastrophe that they are worried about and to rate how awful they believe the catastrophe would be. They are then encouraged to rationally think about how likely it is that the catastrophe would actually happen, how awful it would be if it did happen and, supposing the worst would happen, what would they do to cope. They are also encouraged to fill out what positive and reassuring things they want to say to themselves about the catastrophe now. The exercise promotes cognitive restructuring and the elaboration of balanced responses [62, 63].	1.00 (0.00)	3.00 (0.00)	1.00 (0.75)
7. Modifying rules and assumptions	The exercise can be used to explore participants’ assumptions (a stage of cognition between core beliefs and automatic thoughts), which can be dysfunctional. During the exercise, participants explore the origins, advantages and disadvantages of a rule or assumption. The participants are then encouraged to make adjustments and generate a more flexible alternative rule [64].	1.00 (0.00)	2.50 (1.00)	1.00 (0.75)
8. Belief driven formulation	This exercise is a core belief-driven cognitive behavioral case conceptualization. Such formulations can help to illustrate the critical role of underlying beliefs (i.e., how do their core beliefs influence their thoughts, feelings and behaviors in specific situations). As a result, participants gain an insight into how beliefs can bias their perception of situations, which, in turn, motivates and informs schema change (e.g., [65, 66]).	1.00 (0.00)	3.00 (0.75)	1.00 (0.00)
9. Positive belief record	This exercise is generally used as a schema change technique. During the exercise, participants identify unhelpful core beliefs and formulate a more positive alternative. They are then asked to write down specific examples which support the new belief. This helps individuals to reduce the impact of negative core beliefs while strengthening positive ones [60, 67].	1.00 (0.75)	2.50 (1.00)	1.00 (0.75)
10. Alternative action formulation	This exercise represents a specific version of functional analysis. Participants are encouraged to describe a particular situation and then write down their thoughts, emotions, and behaviors in that situation. In the next step, they are asked to think about alternative responses to the situation: what else could they think in response to the situation, what would they feel if they had these thoughts instead of the actual ones, and what would their behavior be in this case. The exercise helps participants develop more functional responses in terms of thoughts, emotions and behaviors and choose more appropriate coping strategies (adapted from [65, 66, 68]).	1.00 (0.00)	3.00 (0.00)	1.00 (0.00)

#### Intervention design

Both interventions can be classified as low-dose interventions, as they will only last 6 weeks as opposed to more typical 8 weeks programs [26]. However, it has previously been shown that interventions, which are as short as 2 weeks (and are thus more adapted to suit employees with busy work schedules and high workload), can have positive effects on outcomes such as emotional exhaustion [20]. The interventions will combine two types of practice, with each week consisting of two audio-guided active sessions (Mondays and Thursdays; about 30 min each) and home assignments, which will be accompanied by handouts and worksheets (Tuesdays, Wednesdays, Fridays, Saturdays; about 15 min each day).

The first and the last session will serve as an introductory and a closing session, respectively, and will be purely psychoeducational. The main focus of the first session will be on introducing the intervention as well as on increasing the knowledge of core concepts of CBT or mindfulness (i.e., explaining the theoretical framework of the intervention). The last session, on the other hand, will be prepared in such a way that it encourages the participants to continue using the techniques they have learned and that it facilitates the transfer of the newly acquired knowledge into everyday life. In contrast, the central ten sessions will be built around the exercises selected in the preliminary study, ordered in such a way that participants first start with simple and basic exercises and then gradually build upon them (in mindfulness by expanding on the initial experience with the body scan and bringing awareness to the present moment; in CBT by expanding on the initial realization that thoughts are not facts and the ABC model). During the audio-guided session, participants will be taught psychoeducational elements that are specifically relevant for the given exercise and will be guided through their first experience performing the exercise. They will then be encouraged to repeat the exercises on their own in the days that follow, using handouts and worksheets to guide and record their progress. The content of some of the exercises (in both interventions) will be adapted for the working context.

### Measures

All four questionnaire batteries (pre-screening, T1, T2, T3) will include measures of burnout and psychological detachment (primary outcomes). In the pre-screening questionnaire, these two constructs will be accompanied by additional questions aimed at collecting data on background variables (e.g., demographic data). The questionnaire battery, which will be administered at T1, T2, and T3, will also include the following constructs: quality of sleep, work engagement, workaholism, work-family conflict, positive and negative affect, life satisfaction, depression, anxiety, stress (secondary outcomes), mindfulness, work-related maladaptive thinking patterns (potential mechanisms of change), supervisor support for recovery, segmentation supplies, unfinished tasks and time pressure (potential moderators). Furthermore, some additional background variables related to the evaluation of the intervention will be asked at T2 (directly after the intervention, e.g., overall satisfaction with the intervention).

### Work-specific outcomes

#### Burnout

Burnout will be measured using the Maslach Burnout Inventory - General Survey [71], which consists of 16 items that measure three burnout dimensions – professional efficacy (e.g., “*I can effectively solve the problems that arise in my work*”), exhaustion (e.g., “*I feel emotionally drained from my work*”) and cynicism (e.g., “*I’ve become less interested in my work since I started this job*”). All items are answered on a 7-point Likert scale from 0 (never) to 6 (every day). The professional efficacy (α = .71), cynicism (α = .79) and emotional exhaustion (α = .90) subscales all show satisfactory internal consistency [71].

#### Psychological detachment

Psychological detachment will be measured with the psychological detachment subscale of the Recovery Experience Questionnaire developed by Sonnentag and Fritz [72]. The subscale consists of 4 items (e.g., “*I forget about work”*). Participants are asked to rate their agreement with each item on a five-point agreement scale ranging from 1 (strongly disagree) to 5 (strongly agree). In the original study, Cronbach’s alphas for the psychological detachment subscale ranged from .84 to .85 [72].

#### Quality of sleep

Participants will also be asked to fill out the Pittsburgh Sleep Quality Index (PSQI [73];. While the PSQI normally consists of 10 items, we will omit the last one as it does not contribute to the total score. The response format varies; some questions (e.g., “*During the past month, what time have you usually gone to bed at night?*”) ask participants to provide short answers, while the majority of items are answered using a 4-point Likert scale. In scoring the index, seven component scores are derived (i.e., subjective sleep quality, sleep latency, sleep duration, sleep efficiency, sleep disturbance, use of sleep medication, and daytime dysfunction), which can also be summed up to into a global score that shows good internal consistency (α = .80 [74]).

#### Work engagement

Work engagement will be measured using the 9-item Utrecht Work Engagement Scale [75]. The scale consists of three subscales: vigor (e.g., “*At my work, I feel bursting with energy*”; α = .77), dedication (e.g., “*I am enthusiastic about my job*”; α = .85) and absorption (e.g., “*I feel happy when I am working intensely*”; α = .78). The items can also be added up into an overall work engagement score (α = .92 [75]). Participants are asked to indicate how often they had felt as described in the items by choosing the number from 1 (never) to 6 (always).

#### Workaholism

Participants will complete the 10-item Dutch Workaholism Scale [76], which measures two dimensions – working excessively (e.g., “*I seem to be in a hurry and racing against the clock*”) and working compulsively (e.g., “*It is important to me to work hard even when I don’t enjoy what I’m doing*”). All items are answered on a 4-point frequency scale from 1 (almost never) to 4 (almost always). Both dimensions have acceptable internal consistency (α = .78 [76]).

#### Work-family conflict

Work-family conflict will be measured using the Work-Family Conflict Scale [77]. While the scale generally consists of 18 items, which measure 6 dimensions (time-based, strain-based, and behavior-based interference in both directions - work to family and family to work), we will only use 6 items that refer to time-based work interference with family (time-based WIF) and strain-based WIF. Example items are: “*My work keeps me from my family activities more than I would like*” (time-based WIF) and “*When I get home from work I am often too frazzled to participate in family activities/responsibilities*” (strain-based WIF). The answers are given on a 5-point scale ranging from 1 (disagree strongly) to 5 (agree strongly). The reliabilities of both subscales exceed the conventional levels of acceptance and range from .85 to .87 [77].

### General well-being outcomes

#### Positive and negative affect

Positive and negative affect will be measured with the 10-item International Positive and Negative Affect Schedule Short Form [78]. Both positive affect (PA; e.g., “*Inspired*”) and negative affect (NA; e.g., “*Upset*”) are measured with five adjectives. The respondent is asked to express to what extent he/she normally feels as described by the adjective, to which he/she responds with a 5-point frequency scale from 1 (never) to 5 (always). Both subscales have previously shown adequate reliability (PA: α = .78; NA: α = .76 [78]).

#### Life satisfaction

The Satisfaction With Life Scale [79] will be used to assess life satisfaction. The scale consists of 5 items (e.g., “*I am satisfied with my life*”), which are answered on a 7-point agreement scale ranging from 1 (strongly disagree) to 7 (strongly agree). The scale exhibits appropriate internal consistency (α = .87 [79]).

#### Depression, anxiety, stress

Participants will be asked to complete The Depression, Anxiety and Stress Scale - 21 Items (DASS-21 [80];). Each of the three subscales (depression, anxiety, and stress) consists of 7 items, which are answered on a 4-point scale from 0 (did not apply to me at all) to 3 (applies to me very much, or most of the time). A sample item for depression is: “*I couldn’t seem to experience any positive feeling at all*”. A sample item for anxiety is: “*I experienced breathing difficulty*”. A sample item for stress is: “*I found it hard to wind down*”. In one of the validation studies, Cronbach’s alphas for the DASS-21 subscales were .94 for depression, .87 for anxiety, and .91 for stress [81].

### Potential mechanisms

#### Mindfulness

Based on a review of mindfulness measures [82], we decided to use The Mindfulness Attention and Awareness Scale (MAAS [83]). MAAS is comprised of 15 items that measure mindfulness as a trait and five items that measure mindfulness as a state. In our study, only the trait items will be used (e.g., “*I could be experiencing some emotion and not be conscious of it until some time later*”), which are answered on a 6-point scale ranging from 1 (almost always) to 6 (almost never). The Cronbach’s alpha in the validation study was .87 [83].

#### Work-related maladaptive thinking patterns

Work-related maladaptive thinking patterns will be measured using the Work-Related Maladaptive Thinking Patterns Questionnaire [84]. The questionnaire is formative in nature and consists of 15 items. Each item refers to one cognitive distortion that can arise in the work context (e.g., unfair comparisons: “*My colleagues are faster and more skillful than me*”). Participants are asked to indicate how likely it is for them to think in the same way as described in the items, to which they respond using a 7-point scale from 1 (extremely unlikely) to 7 (extremely likely). The questionnaire has previously exhibited great internal consistency, ranging from .86 to .87 [84].

### Potential moderators and control variables

#### Supervisor support for recovery

Supervisor support for recovery will be measured using the 6-item Supervisor Support for Recovery Scale [35]. The scale was originally designed to be filled out by the supervisor. However, we will adapt the items so they can be answered by the employee, shifting the emphasis to the employee’s perception of the supervisor’s support for recovery (e.g., instead of “*I expect my subordinates to be willing to work around the clock, if necessary”*, this version will be used: “*My immediate supervisor expects his/her subordinates to be willing to work around the clock, if necessary”*). All responses are made on a 5-point scale from 1 (strongly disagree) to 5 (strongly agree). In the validation study, the scale has exhibited great internal consistency (α = .90 [35]).

#### Work-home segmentation supplies

Participants will also be asked to complete the 4-item Segmentation supplies scale developed by Kreiner [36]. All items are answered using a 7-point agreement scale from 1 (strongly disagree) to 7 (strongly agree), with 4 being neutral. An example item is: “*My workplace lets people forget about work when they’re at home*”. The scale has great internal consistency (α = .94 [36]).

#### Unfinished tasks

Unfinished tasks will be measured with items developed by Syrek, Weigelt, Peifer, and Antoni [37]. While the original version of the scale consists of 6 items, we will use a shorter 3-item version based on our previous work [85]. An example item is: “*I do not even start with important tasks I want to fulfill*”. The response scale ranges from 1 (strongly disagree) to 5 (strongly agree). The 3-item version has great internal consistency, with Cronbach’s α = .88 [85].

#### Time pressure

Lastly, time pressure will be measured using the adapted version of the ISTA (Instrument for stress-related job analysis) “time pressure” subscale [86, 87]. The subscale is comprised of 3 items (e.g., “*I was pressed for time*”), answered on a 5-point agreement scale ranging from 1 (strongly disagree) to 5 (strongly agree). The scale has previously shown acceptable internal consistency (α = .83 [86]).

#### Background variables

In the pre-screening questionnaire, participants will be asked to provide some basic demographic data (i.e., gender, age, the highest level of formal education and family status), basic information regarding their work (i.e., employment status, type of work, weekly working hours, tenure, leadership status and whether they are currently on sick leave) as well as some intervention-related data (i.e., willingness to dedicate enough time to the intervention, access to the internet, self-rated competency to use a computer, involvement in other forms of psychotherapy/psychoeducation, prescription of drugs to treat mental illnesses and the presence of a mental illness diagnosis).

In the questionnaire that will be administered at T2 (directly after the intervention), they will additionally be asked to report any significant work- (e.g., switching jobs), private life- (e.g., switching apartments), or intervention-related (e.g., participating in other interventions, aimed at improving mental health) changes during the last 6 weeks. They will also be asked to rate their overall satisfaction with the intervention, their attitudes towards the exercises, their previous experience with similar exercises, and their self-reported adherence to the intervention procedure.

### Statistical analysis

#### Sample size calculation

The G*Power 3.1 software was used to determine the minimum number of participants that need to be recruited [64]. We set the expected effect size to medium (*f(V)* = .25), the probability of a Type I error to α = .05, the statistical power (1-β) to .95 and chose a two-way repeated measures MANOVA (multivariate analysis of variance) with time (3 levels: pre, post, and follow-up) as the within-subjects factor and group (3 levels: CBT, MBSR, and control) as the between-subjects factor as the main statistical test. Given these specifications, the required total sample size is 151 participants (approximately 50 participants per group). In case of non-adherence and/or drop-out, the total sample size of 98 participants (approximately 33 participants per group) suffice with a more standard power level of .80.

#### Effect evaluation

The analyses will be carried out with SPSS [88] and Mplus [89]. The significance level used in the analyses will be .05. The main research questions (i.e., group differences in psychological detachment from work and burnout) will be analyzed with two separate two-way repeated measures analyses of variance (ANOVAs), with time as the within-subjects factor (3 levels: pre, post, follow-up) and group as the between-subjects factor (3 levels: CBT, MBSR, control), for each of the main outcomes separately. Significant interactions will be followed up with a simple effects’ analysis, using the Bonferroni correction to adjust for multiple testing.

The effects of the intervention on the secondary outcomes (i.e., general and work-related well-being) will be analyzed with a two-way repeated-measures MANOVA, with time as the within-subjects factor and group as the between-subjects factor. This analysis is contingent upon moderate correlations between the dependent variables [90]. Therefore, we will first examine correlations between the secondary outcome variables. Significant interaction effects will be followed-up with ANOVAs for each of the dependent variables and a discriminant descriptive analysis to further examine the effects. The moderation (i.e., supervisor support for recovery, work-home integration supplies, unfinished tasks, time pressure) and mediation (i.e., mindfulness, work-related maladaptive thinking patterns) effects will be analyzed using the PROCESS macro for SPSS [91]. T2 measures of the studied mediators will be utilized in these analyses. In order to explore both; the inter-individual variability and the intra-individual patterns of change trajectories in the mechanisms and the main outcomes during interventions, growth curve analyses will be conducted using data from the weekly questionnaires.

## Discussion

Many employees find it hard to disengage from work during non-work time. Research largely found that this is not without costs with respect to well-being. In responding to this salient issue, the paper describes a protocol for a randomized control trial aimed at enhancing psychological detachment from work and employee well-being. More precisely, the article provides a basis for the development and design of a study that enables a direct comparison of the effectiveness of online CBT-based and mindfulness interventions and the assessment of mechanisms of change. Additionally, the paper highlights different factors that are likely to determine the strength of the intervention effects. As such, the study has the potential to extend the knowledge of evidence-based interventions in the workplace context and their refinement. By applying the medical *ex juvantibus* logic (i.e., proposing a diagnosis based on the observed response of the disease to a treatment), the findings of the study will further help to make inferences about the underlying theoretical mechanisms responsible for poor psychological detachment from work and work-related ill-being.

### Strengths and limitations

The proposed study, however, is not without its limitations. First, a more general limitation is related to the focus of the intervention. The intervention can be considered as an individual-focused intervention aimed at teaching employees how to better deal with demanding work conditions and, in turn, become more relaxed and more resilient. As the culture, norms, and practices in many organizations today encourage constant availability and extended work hours [92], some of the underlying causes of poor psychological detachment and ill-being thus will not be tackled. In order to deal with these organizational issues, organization-focused interventions that would result in meaningful changes in the work environment, lower levels of job demands, and increased resources (i.e., job re-design) should also be considered [15]. Moreover, multimodal interventions combining individual- and organization-focused elements should be sought after, as they are most likely to result in sustained beneficial effects for employee well-being [93]. Second, both the CBT and mindfulness interventions are based on a pre-defined set of online exercises and homework, which may undermine the participants’ need for autonomy. Previous studies have shown that workplace interventions should offer participants the opportunity to form their own intervention objectives and exercises during the intervention process [94]. Such a participative design is likely to facilitate intervention effects by being tailored to employees’ specific needs and preferences. Nevertheless, it should be considered that our online interventions provide a high degree of flexibility in terms of time and location of practicing mindfulness and carrying out the CBT exercises and homework. Third, the exercises and homework can take up a significant amount of time. Employees may eventually become less motivated or even fatigued by the added burden to their daily routines, which can attenuate the intervention effects, lead to non-adherence, or even drop-out. Selective drop-out in the CBT intervention group may be particularly problematic, as CBT homework may be perceived as more difficult than mindfulness exercises [33]. The online intervention design can also represent a potential factor of non-adherence and drop-out, as employees may feel less obliged to participate in the protocol than in face-to-face meetings. In line with previous interventions in the workplace context [95], several measures will be taken to avoid these complications. Participants will be briefed about the duration of each of the sessions and the overall intervention in an informed consent form. We will also stress the importance of participating in the study throughout the whole intervention. In-between sessions, participants will also receive e-mail reminders. Adherence will also be monitored by the online platform, which will additionally help to determine whether one of the groups is more compliant with the protocol than the other.

Notwithstanding these limitations, the proposed intervention study is one of the first that is explicitly directed at comparisons between two protocols and mechanisms of change. Both notions are important, as the results may inform theory about psychological detachment and lead to the identification of decisive intervention ingredients. With the focus on work-related maladaptive thinking patterns [40, 84], the work-related well-being literature will potentially gain understanding of a new yet poorly understood, personal vulnerability factor that makes employees more susceptible to ill-being at work or even psychological disorders. Another strength of the intervention is the control of relevant confounding factors. Although the online intervention design may have some drawbacks, the contamination of experimental and control groups will not be a cause for concerns. As participants will not be recruited from one or a small number of organizations and will individually apply for the study, exchanges between participants from different intervention groups are not likely. Given the online protocol, intervention effects also cannot be attributed to positive and supportive interactions between participants [14]. As participants will be blinded to the intervention, the possibility of attenuated or accentuated intervention effects due to participants’ expectations will be minimal. Finally, both online interventions can be utilized by a wide range of professions and can easily be adapted to different workplace contexts and work schedules. The weekly monitoring of effects will further help to customize and shorten similar interventions in the future. As such, the protocol has the potential to significantly advance knowledge about evidence-based CBT and mindfulness interventions and holds promise to be highly applicable to organizational settings.

## Data Availability

Anonymized raw data will be shared with researchers upon request by contacting the corresponding author (after completion of the research project).

## Abbreviations

CBT: Cognitive-Behavioral Therapy
MBSR: Mindfulness-Based Stress Reduction
MBCT: Mindfulness-Based Cognitive Therapy
CONSORT: Consolidated Standards of Reporting Trials
PSQI: Pittsburgh Sleep Quality Index
WIF: Work Interference With Family
PA: Positive Affect
NA: Negative Affect
DASS-21: The Depression, Anxiety and Stress Scale - 21
MAAS: The Mindfulness Attention and Awareness Scale
ISTA: Instrument for Stress-Related Job Analysis
MANOVA: Multivariate Analysis of Variance
ANOVA: Analysis of Variance

## References

[1] Eurofound (2017). Working anytime, anywhere: The effects on the world of work.

[2] Eurofound (2016). Sixth European working conditions survey – overview report.

[3] Eurofound (2018). Striking a balance: Reconciling work and life in the EU.

[4] Schaufeli W (2018). Burnout in Europe: Relations with national economy, governance, and culture. KU Leuven. Research Unit Occupational and Organizational Psychology and Professional Learning (internal report).

[5] Hassard J, Teoh KRH, Visockaite G, Dewe P, Cox T (2018). The cost of work-related stress to society: a systematic review. J Occup Health Psychol.

[6] Meijman TF, Mulder G, Drenth P, Thierry H, de Wolff C (1998). Psychological aspects of workload. Handbook of work and organizational psychology.

[7] Etzion D, Eden D, Lapidot Y (1998). Relief from job stressors and burnout: reserve service as a respite. J Appl Psychol..

[8] Sonnentag S (2018). The recovery paradox: portraying the complex interplay between job stressors, lack of recovery, and poor well-being. Res Organ Behav.

[9] Sonnentag S, Fritz C (2015). Recovery from job stress: the stressor-detachment model as an integrative framework. J Organ Behav.

[10] Querstret D, Cropley M (2012). Exploring the relationship between work-related rumination, sleep quality, and work-related fatigue. J Occup Health Psychol.

[11] Wendsche J, Lohmann-Haislah A (2017). A meta-analysis on antecedents and outcomes of detachment from work. Front Psychol.

[12] Bennett AA, Bakker AB, Field JG (2018). Recovery from work-related effort: a meta-analysis. J Organ Behav.

[13] Hahn VC, Binnewies C, Sonnentag S, Mojza EJ (2011). Learning how to recover from job stress: effects of a recovery training program on recovery, recovery-related self-efficacy, and well-being. J Occup Health Psychol.

[14] Bartlett L, Martin A, Neil AL, Memish K, Otahal P, Kilpatrick M (2019). A systematic review and meta-analysis of workplace mindfulness training randomized controlled trials. J Occup Health Psychol.

[15] Bostock S, Crosswell AD, Prather AA, Steptoe A (2019). Mindfulness on-the-go: effects of a mindfulness meditation app on work stress and well-being. J Occup Health Psychol.

[16] Hülsheger UR, Feinholdt A, Nübold A (2015). A low-dose mindfulness intervention and recovery from work: effects on psychological detachment, sleep quality, and sleep duration. J Occup Organ Psychol.

[17] Querstret D, Cropley M, Fife-Schaw C (2017). Internet-based instructor-led mindfulness for work-related rumination, fatigue, and sleep: assessing facets of mindfulness as mechanisms of change. A randomized. J Occup Health Psychol.

[18] Querstret D, Cropley M, Kruger P, Heron R (2016). Assessing the effect of a cognitive behaviour therapy (CBT)-based workshop on work-related rumination, fatigue, and sleep. Eur J Work Organ Psychol.

[19] Brenninkmeijer V, Lagerveld SE, Blonk RWB, Schaufeli WB, Wijngaards-de Meij LDNV (2019). Predicting the effectiveness of work-focused CBT for common mental disorders: the influence of baseline self-efficacy, depression and anxiety. J Occup Rehabil.

[20] Hülsheger UR, Alberts HJ, Feinholdt A, Lang JW (2013). Benefits of mindfulness at work: the role of mindfulness in emotion regulation, emotional exhaustion, and job satisfaction. J Appl Psychol..

[21] Leroy H, Anseel F, Dimitrova NG, Sels L (2013). Mindfulness, authentic functioning, and work engagement: a growth modeling approach. J Vocat Behav.

[22] Michel A, Bosch C, Rexroth M (2014). Mindfulness as a cognitive-emotional segmentation strategy: an intervention promoting work-life balance. J Occup Organ Psychol.

[23] Slutsky J, Chin B, Raye J, Creswell JD (2019). Mindfulness training improves employee well-being: a randomized controlled trial. J Occup Health Psychol.

[24] van der Klink JJ, Blonk RW, Schene HA, van Dijik FJ (2001). The benefits of interventions for work-related stress. Am J Public Health.

[25] Allen TD, Eby LT, Conley KM, Williamson RL, Mancini VS, Mitchell ME (2015). What do we really know about the effects of mindfulness-based training in the workplace?. Ind Organ Psychol.

[26] Eby LT, Allen TD, Conley KM, Williamson RL, Henderson TG, Mancini VS (2019). Mindfulness-based training interventions for employees: a qualitative review of the literature. Hum Resour Manag Rev.

[27] Kazdin AE (2007). Mediators and mechanisms of change in psychotherapy research. Annu Rev Clin Psychol.

[28] Fjorback LO, Arendt M, Ornbol E, Fink P, Walach H (2011). Mindfulness-based stress reduction and mindfulness-based cognitive therapy - a systematic review of randomized controlled trials. Acta Psychiatr Scand.

[29] Kabat-Zinn J (1982). An outpatient program in behavioral medicine for chronic pain patients based on the practice of mindfulness meditation: theoretical considerations and preliminary results. Gen Hosp Psychiatry.

[30] Segal ZV, Williams JMG, Teasdale J (2002). Mindfulness-based cognitive therapy for depression: a new approach to preventing relapse.

[31] Hayes SC, Villatte M, Levin M, Hildebrandt M (2011). Open, aware, and active: contextual approaches as an emerging trend in the behavioral and cognitive therapies. Annu Rev Clin Psychol.

[32] Goldin PR, Morrison A, Jazaieri H, Brozovich F, Heimberg R, Gross JJ (2016). Group CBT versus MBSR for social anxiety disorder: a randomized controlled trial. J Consult Clin Psychol.

[33] Hofheinz C, Reder M, Michalak J (2020). How specific is cognitive change? A randomized controlled trial comparing brief cognitive and mindfulness interventions for depression. Psychother Res.

[34] Creswell JD (2017). Mindfulness interventions. Annu Rev Psychol.

[35] Bennett AA, Gabriel AS, Calderwood C, Dahling JJ, Trougakos JP (2016). Better together? Examining profiles of employee recovery experiences. J Appl Psychol..

[36] Kreiner GE (2006). Consequences of work-home segmentation or integration: a person-environment fit perspective. J Organ Behav.

[37] Syrek CJ, Weigelt O, Peifer C, Antoni CH (2017). Zeigarnik's sleepless nights: how unfinished tasks at the end of the week impair employee sleep on the weekend through rumination. J Occup Health Psychol.

[38] Jamieson SD, Tuckey MR (2017). Mindfulness interventions in the workplace: a critique of the current state of the literature. J Occup Health Psychol.

[39] Beck AT, Dozois DJA (2011). Cognitive therapy: current status and future directions. Annu Rev Med.

[40] Van Wijhe C, Peeters M, Schaufeli W (2013). Irrational beliefs at work and their implications for workaholism. J Occup Rehabil.

[41] Querstret D, Cropley M, Fife-Schaw C (2018). The effects of an online mindfulness intervention on perceived stress, depression and anxiety in a non-clinical sample: a randomised waitlist control trial. Mindfulness (N Y)..

[42] Good DJ, Lyddy CJ, Glomb TM, Bono JE, Brown KW, Duffy MK (2016). Contemplating mindfulness at work: an integrative review. J Manage.

[43] Schulz KF, Altman DG, Moher D (2011). CONSORT 2010 statement: updated guidelines for reporting parallel group randomised trials. Int J Surg.

[44] De Bloom J, Kinnunen U, Korpela K (2014). Exposure to nature versus relaxation during lunch breaks and recovery from work: development and design of an intervention study to improve workers' health, well-being, work performance and creativity. BMC Public Health.

[45] Amichai-Hamburger Y, Klomek A, Friedman D, Zuckerman O, Shani-Sherman T (2014). The future of online therapy. Comput Human Behav.

[46] Rochlen AB, Zack JS, Speyer C (2004). Online therapy: review of relevant definitions, debates, and current empirical support. J Clin Psychol.

[47] Carlson L (2013). Mindfulness-based cancer recovery: the development of an evidence-based psychosocial oncology intervention. Oncol Exch.

[48] Aikens K, Astin J, Pelletier KR, Levanovich K, Baase C, Park YY (2014). Mindfulness goes to work: impact of an online workplace intervention. J Occup Environ Med.

[49] Andersson G, Cuijpers P, Carlbring P, Riper H, Hedman E (2014). Guided internet-based vs. face-to-face cognitive behavior therapy for psychiatric and somatic disorders: a systematic review and meta-analysis. World Psychiatry.

[50] Carlbring P, Andersson G, Cuijpers P, Riper H, Hedman-Lagerlöf E (2018). Internet-based vs. face-to-face cognitive behavior therapy for psychiatric and somatic disorders: an updated systematic review and meta-analysis. Cogn Behav Ther.

[51] Ruwaard J, Lange A, Schrieken B, Dolan CV, Emmelkamp P (2012). The effectiveness of online cognitive behavioral treatment in routine clinical practice. PLoS One.

[52] Spijkerman M, Pots W (2016). Review EB-C psychology, 2016 U. Effectiveness of online mindfulness-based interventions in improving mental health: A review and meta-analysis of randomised controlled trials. Clin Psychol Rev.

[53] Vonderlin R, Biermann M, Bohus M, Lyssenko L (2020). Mindfulness-based programs in the workplace: A meta-analysis of randomized controlled trials. Mindfulness (N Y).

[54] Framson C, Kristal AR, Schenk JM, Littman AJ, Zeliadt S, Benitez D (2009). Development and validation of the mindful eating questionnaire. J Am Diet Assoc.

[55] Teasdale J, Williams J, Segal Z (2014). The mindful way workbook: an 8-week program to free yourself from depression and emotional distress.

[56] Sauer-Zavala SE, Walsh EC, Eisenlohr-Moul TA, Lykins EL (2013). Comparing mindfulness-based intervention strategies: differential effects of sitting meditation, body scan, and mindful yoga. Mindfulness (N Y).

[57] Beck AT, Rush AJ, Shaw BF, Emery G (1979). Cognitive therapy of depression.

[58] Ellis A (1957). Rational psychotherapy and individual psychology. J Individ Psychol.

[59] Addis M, Martell C (2004). Overcoming depression one step at a time: the new behavioral activation approach to getting your life back.

[60] Greenberger D, Padesky C (1995). Mind over mood: a cognitive therapy treatment manual for clients.

[61] Leahy R (1996). Cognitive therapy: basic principles and applications.

[62] Ellis A (1962). Reason and emotion in psychotherapy.

[63] Whalley MG (2017). Psychology tools for overcoming panic. Psychology Tools.

[64] Faul F, Erdfelder E, Lang AG, Buchner A (2007). G* power 3: a flexible statistical power analysis program for the social, behavioral, and biomedical sciences. Behav Res Methods.

[65] Persons J (2012). The case formulation approach to cognitive-behavior therapy.

[66] Tarrier N, Johnson J (2015). Case formulation in cognitive behaviour therapy: the treatment of challenging and complex cases.

[67] Padesky CA (1994). Schema change processes in cognitive therapy. Clin Psychol Psychother.

[68] Haynes S, O'Brien W (2003). Principles and practice of behavioral assessment.

[69] Fenn K, Byrne M (2013). The key principles of cognitive behavioural therapy. InnovAiT..

[70] Beck A (1976). Cognitive therapy and the emotional disorders.

[71] Schaufeli WB, Leiter MP, Maslach C, Jackson SE, Maslach C, Jackson S, Leiter M (1996). The Maslach Brunout inventory-general survey. Maslach burnout inventory manual.

[72] Sonnentag S, Fritz C (2007). The recovery experience questionnaire : development and validation of a measure for assessing recuperation and unwinding from work. J Occup Health Psychol.

[73] Buysse DJ, Reynolds C, Monk TH, Berman SR, Kupfer DJ (1989). The Pittsburgh sleep quality index: a new instrument for psychiatric practice and research. Psychiatry Res.

[74] Carpenter JS, Andrykowski MA (1998). Psychometric evaluation of the Pittsburgh sleep quality index. J Psychosom Res.

[75] Schaufeli WB, Bakker AB (2003). Utrecht work engagement scale: preliminary manual.

[76] Schaufeli WB, Shimazu A, Taris TW (2009). Being driven to work excessively hard: the evaluation of a two-factor measure of workaholism in the Netherlands and Japan. Cross-Cultural Res.

[77] Carlson DS, Kacmar KM, Williams LJ (2000). Construction and initial validation of a multidimensional measure of work–family conflict. J Vocat Behav.

[78] Thompson ER (2007). Development and validation of an internationally reliable short-form of the positive and negative affect schedule (PANAS). J Cross-Cult Psychol.

[79] Diener E, Emmons RA, Larsem RJ, Griffin S (1985). The satisfaction with life scale. J Pers Assess.

[80] Lovibond PF, Lovibond SH (1995). The structure of negative emotional states: comparison of the depression anxiety stress scales (DASS) with the Beck depression and anxiety inventories. Behav Res Ther.

[81] Antony MM, Bieling PJ, Cox BJ, Enns MW, Swinson RP (1998). Psychometric properties of the 42-item and 21-item versions of the depression anxiety stress scales in clinical groups and a community sample. Psychol Assess.

[82] Qu YE, Dasborough MT, Todorova G (2015). Which mindfulness measures to choose to use?. Ind Organ Psychol.

[83] Brown KW, Ryan RM (2003). The benefits of being present: mindfulness and its role in psychological well-being. J Pers Soc Psychol.

[84] Tement S, Plohl N, Noja A, Kubicek B (2020). Work-related maladaptive thinking patterns: theoretical conceptualization and scale development.

[85] Noja A, Kubicek B, Plohl N, Tement S (2020). Development and validation of the work-home-integration questionnaire (WHIQ).

[86] Binnewies C, Sonnentag S, Mojza EJ (2009). Daily performance at work: feeling recovered in the morning as a predictor of day-level job performance. J Organ Behav Int J Ind Occup Organ Psychol Behav.

[87] Semmer N, Zapf D, Dunckel H, Dunckel H (1999). Instrument zur stressbezogenen Tätigkeitsanalyse (ISTA). Handbuch psychologischer Arbeitsanalyseverfahren.

[88] Corp IBM (2016). IBM SPSS statistics for windows, version 24.0.

[89] Muthén L, Muthén B (2007). Mplus statistical software.

[90] Warne RT (2014). A primer on multivariate analysis of variance (MANOVA) for behavioral scientists. Pract Assessment, Res Eval.

[91] Hayes AF (2017). Introduction to mediation, moderation, and conditional process analysis: a regression-based approach.

[92] Kubicek B, Tement S (2016). Work intensification and the work-home interface: the moderating effect of individual work-home segmentation strategies and organizational segmentation supplies. J Pers Psychol.

[93] Awa WL, Plaumann M, Walter U (2010). Burnout prevention: a review of intervention programs. Patient Educ Couns.

[94] Le Blanc PM, Hox JJ, Schaufeli WB, Taris TW, Peeters MC (2007). Take care! The evaluation of a team-based burnout intervention program for oncology care providers. J Appl Psychol.

[95] Kosenkranius MK, Rink FA, De Bloom J, Van Den Heuvel M (2020). The design and development of a hybrid off-job crafting intervention to enhance needs satisfaction, well-being and performance: a study protocol for a randomized controlled trial. BMC Public Health.

